# Investigatingthe Effect of Pressure on Third Liver Point on Primary Dysmenorrhea: a Randomized Controlled Clinical Trial

**DOI:** 10.5812/ircmj.12719

**Published:** 2013-09-05

**Authors:** Mahboobeh Kafaei Atrian, Fatemeh Abbaszadeh, Malihe Sarvieh, Nahid Sarafraz, Mohammad Asghari Jafarabadi

**Affiliations:** 1 Department of Midwifery, Kashan University of Medical Sciences, Kashan, IR Iran; 2 Traffic Injury Prevention Research Center, Department of Statistics and Epidemiology, Faculty of Health, Tabriz University of Medical Sciences, Tabriz, IR Iran

**Keywords:** Acupressure, Dysmenorrhea, Liver

## Abstract

**Background:**

Primary dysmenorrhea (PD) is a term used to describe uterine muscle spasms which occurs during the days of menstruation.

**Objectives:**

To determine the effect of acupressure on third liver point on primary dysmenorrhea.

**Patients and Methods:**

Female students living in dormitories of Kashan University of Medical Sciences in Iran who had PD were studied for three menstrual cycles between March till June 2012. Individuals with depression score higher than 19 according to the Beck-21 Depression scale were excluded. In the first cycle, pain intensity was assessed without intervention, and 67 samples with a pain score greater than 4 according to the visual analogue scale (VAS) were selected. Then they were randomized into third liver point (liv3) and control (placebo) groups using randomized block design with 1:1 allocation ratio based on pain intensity. In the second and third cycles, pressure was applied by the research unit intermittently for 16 minutes (2 minutes pressure, 2 minutes resting) with the starting of blood flow. Primary outcome of this study was the pain intensity which was compared between first and third cycles. Someone who divided groups, samples and data analyzer was blinded.

**Results:**

In the treatment group 27 samples and in the control group 32 samples were analyzed. Friedman test showed significant differences in pain intensity before and after the intervention within both groups (P < 0.05). There were no significant differences between the groups according to the ordinal regression test in 3 cycles (P > 0.05).

**Conclusions:**

The pressure on the LIV3 applied in this investigation was effective in reducing primary dysmenorrheal pain. So using this method is recommended to reduce PD.

## 1. Background

Primary dysmenorrhea is a term used to describe uterine muscle spasms which occurs during the days of menstruation (1). Age at menarche, weight, menstrual intervals, duration of bleeding flow, and family history are factors affecting PD (2-9). Its prevalence has been reported from 1.7% to 97% (10). Currently analgesics, hormones in contraceptive pills and nonsteroidal anti-inflammatory drugs are used in the treatment of PD. But they have many adverse effects. A study reported 42389 serious adverse drug reactions during 2002-2006 years in France (11). So there is a tendency to find a relief for PD without side effects. Acupressure is used in the treatment of PD in traditional Chinese medicine (TCM). Acupressure balances the flow of energy (chi) in the body (10). “Effectiveness of pressure on Sanyinjiao” or “spleen sixth” (SP6) points in relief of PD has been reported previously (12-17), but we found only one study about third liver or Taichong (liv3) point. Bazarganipour et al. (2010) study showed that pressure on this point is a simple, inexpensive and very effective way for reducing the pain of PD (18). At all studies in this area are insufficient and further studies are suggested (10, 16, 19).

## 2. Objective

Considering that acupressure is a simple and inexpensive way for reducing pain, and general lack of studies in this area, especially in the third liver point, in this study we intended to determine the effect of acupressure on third liver point on primary dysmenorrhea.

## 3. Patients and Methods

### 3.1. Trial design

**Figure 1.**
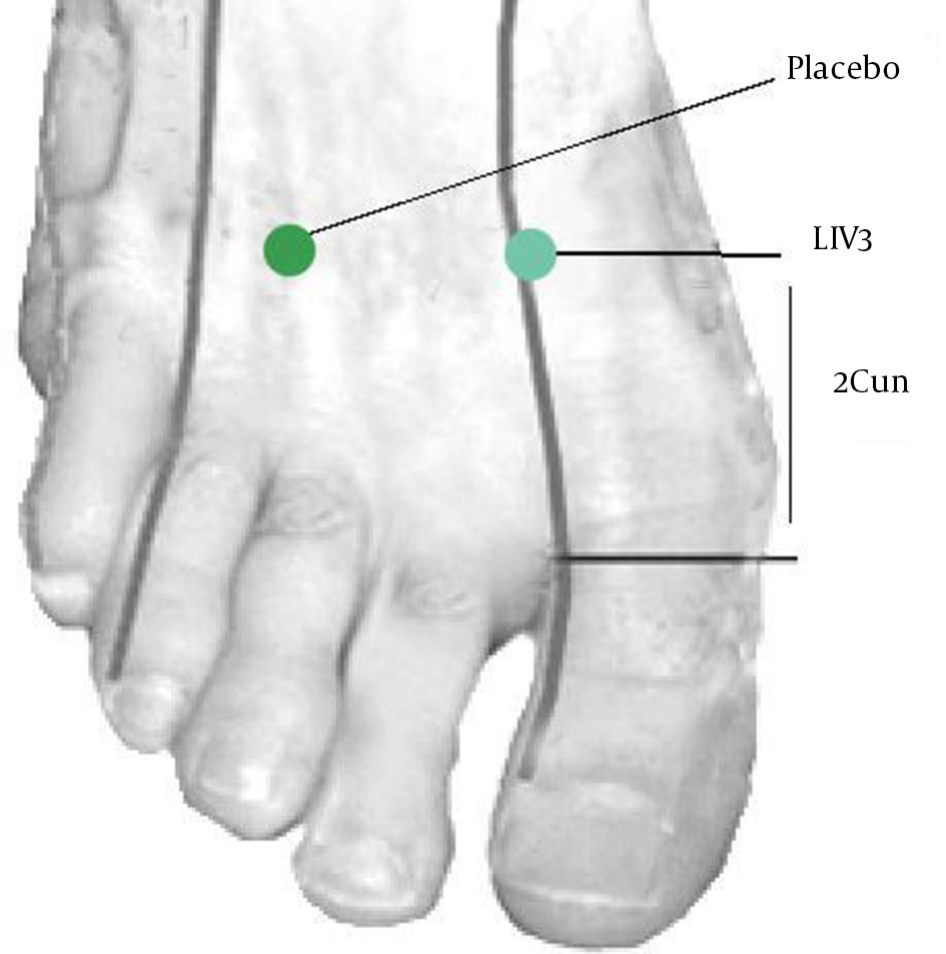
Liv3 and Placebo Points

This is a clinical trial performed in the treatment and placebo groups registered in the Iranian Registry of Clinical Trials (IRCT). Its Registration ID is: IRCT201201308869N1. Liv3 point, is located 2 cm (Width of three fingers) above the distance between the ﬁrst and second metatarsal bones and the placebo point was located 2 cm above the distance between the third and fourth fingers (Figure 1) (18). 

### 3.2. Participants

Female students living in dormitories of Kashan University of Medical Sciences-Iran with PD were studied for three menstrual cycles between March till June 2012. Inclusion criteria were: being a student at dormitory, being single, regularity of menses, start of pain with the onset of menstrual bleeding, duration of bleeding between 3-8 days and menstrual intervals of 21-35 days, pain with a score of at least 4 of 10 according to the VAS criteria in most menstrual cycles, lake of pain throughout all times of menstrual cycle or bleeding, lake of anemia, high blood pressure, psychiatric disorders, especially depression (more than 19 point according to the beck-21 criteria for depression), lake of any known disease of genital tract, secondary dysmenorrhea, no history of abdominal or pelvic surgery, not using tobacco (cigarettes, hookah and drugs) and alcohol, disorders of speech and hearing, mental, heart and renal disorders, respiratory disease, diabetes, asthma, hypothyroidism or hyperthyroidism, severe psychological stress in the past 6 months (e.g., family death, surgery, marriage, separation of parents), lake of voluntary weight loss, absence of any problems in the pressure point such as fractures, ulcers, varicose veins, skin disease or inflammation, specific dietary regimen such as vegetarianism, eating raw, high salt or carbohydrates intake. Exclusion criteria were: use of heat, oral contraceptives or drugs that can affect ovulation cycle, nonsteroidal anti-inflammatory, analgesic, prostaglandins synthesis inhibitors for 4 hours before till 4 hours after applying pressure, and unwillingness to cooperate until the end of the study (3 cycles). Finally 32 participants in control group and 27 in trial group were analyzed.

Sample size: In the Kashefiet al. (2010) study, the average pain intensity in the second hour after intervention in the treatment and control groups were 4.55 ± 1.60 and 6.34 ± 1.57, so according to the following formula: N=[Z(1-α/2)+Z(1-β)] 2 (S1^2^+S2^2^)/(μ_1_-μ_2_)^2^ with 90% power and 95% confidence interval and regarding loss of 30% of the sample size, a minimum sample size of 23 samples per group was needed. Regarding the inclusion and exclusion criteria and the possible loss of the samples, 500 students living in the residence were invited for participation. The only observer was the third author of this article who was midwifery student and performed sampling. She has been in constant contact with the subjects face to face, by telephone or by post. Also she controlled proper technic of pressure that was applied by samples. Initially, 104 samples fulfilled the inclusion criteria, received explanation about acupressure and informed consent was obtained. Participants completed the Beck-21 depression scale to exclude people who had depression score > 19 (20), and were received a questionnaire containing demographic and menstrual cycle information. Pain intensity was measured on the first day of first cycle without intervention using VAS. At this time 59 samples with a pain score of ≥ 4 remained to participate in the study.

### 3.3. Randomization

Subjects were divided into two parallel groups, including group a (placebo point) and group b (third liver point), using a randomized block allocation (allocation ratio 1:1) based on pain intensity.

### 3.4. Blinding

Group division was determined by someone who was unaware of the experimental groups using a random number that was conducted by demographer. The research unit and data analyzer were not aware of intervention and control groups. Intervention method was similar in both groups. This type of blinding was the strong point of this study.

### 3.5. Interventions

The first researcher was received training in acupressure from the TCM professor. The subjects were instructed to apply pressure techniques and find the exact location of pressure points. Acuhealth device was used to ensure the accuracy of pressure points and the Force Gauge was used for unification of pressure. Firstly, pressure was applied on acupoints and continued until research unit announce De chi (i.e. Feeling of tingling, heat, cold, creep). At this time the pressure showed by the gauge’s screen was recorded. Then the subjects were asked to apply the same pressure and pay attention to their nail color changes. After then they made a pressure till the same color change was occurred. Pressure was applied twice on each leg and a total of four times (16 minutes) in the clockwise rotation alternatively. Each time the pressure would stop with the sense of De chi, otherwise continued for 2 minutes and was resumed after 2 minutes on the other foot. In the second and third cycles, pressure was exerted by the participants at the start of bleeding. The pain was measured immediately and 0.5, 1, 2, 3 and 4 hours after the start of bleeding by research unit using VAS.

### 3.6. Outcomes

The primary outcome was the pain which was assessed before and after the start of bleeding using the VAS scale and were compared before and after intervention.

### 3.7. Instruments

The Force Gauge device determines the pressure applied by the researcher finger. This device is made in Taiwan and has the international standard of the European Union and valid certificate of calibration ISO 9001 (21). Australian acuhealth professional 900 devices were used. It makes different sound to find the correct acupressure point. This device is approved by the American Food and Drug Administration and has approval of the Iranian Ministry of Health and Medical Education (21). This device has been used in several studies and its reliability has been confirmed previously (21). Made in German digital glass scale (GS46) with 100 grams accuracy was used to measure the weight, and a single non elastic tape was used to measure the height.

The Beck-21 depression inventory for adults, have 21 groups of questions that each group would receive a score of zero to 3, and a total of 63 points. Overall rating scale of <10 indicate no or minimal depression, 10 to 18 indicate mild to moderate depression, 19 to 29 indicate moderate to severe depression, and 30 to 63 indicate severe depression(20). The validity of this inventory has been approved previously (20, 22), and its reliability was reviewed and confirmed in this study (Cronbach's alpha equal to 0.887 > 0.7). The VAS pain scale questionnaire is a ruler which research unit marked it upon her pain. Distance from the beginning of the ruler to marked point in centimeter would be the pain Score of the unit (1, 12). The VAS is reliable and valid for subjective experiences including pain (12). Also the content validity of the questionnaire was determined by a panel of 10 expert person including MS in Midwifery, and MS in Nursing and obstetricians. As well its reliability was reviewed and confirmed in this study (Cronbach's alpha equal to 0.862 > 0.7).

### 3.8. Analysis

Data was analyzed using SPSS 13 software (SPSS, Inc. I1 Chicago, The USA). Confounding variables (painful menstrual history in first degree relatives, age at menarche, interval between menarche and onset of PD, menstrual duration and BMI) were equalized in both groups. Data was reported with a mean ± SD for quantitative, frequency (%) for qualitative and median interquartile range (IQR) for pain ordinal variables. Mann-Whitney test was used to compare the intensity of pain in the two groups. Mann-Whitney test or chi-square test was used to check the association of the underlying quantitative, ordinal or nominal variables respectively in groups according to the nature of the variables, and confounding variables were adjusted using ordinal regression analysis if they were not matched. Sign test was used to compare pain intensity in the first and third cycles within each group. In case of significant results post hoc test with Bonferroni correction was used to assess the pair wise comparisons. Ordinal regression analysis was used to compare pain intensity between groups in the third cycle. P < 0.05 was considered statistically significant.

## 4. Results

**Figure 2.**
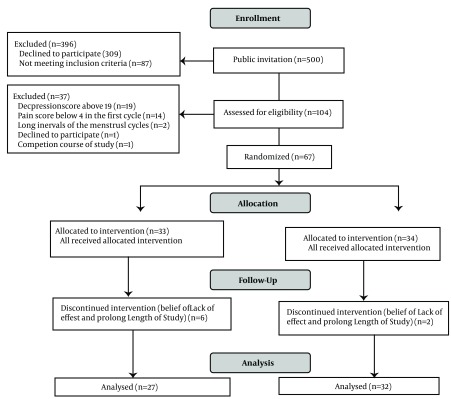
Flow Diagram

Among the volunteers of public invitation from 500 students living at the hostel, regarding inclusion and exclusion criteria, 59 samples with pain score≥ 4 were remained to be analyzed. Loss of the samples is shown in Figure 2. 

The students were studying at different fields of medical sciences. Comparing the characteristics of the two groups in the first cycle is shown in Table 1. Pearson Chi-Square test showed that there were no significant differences between groups for courses and family history of PD. Results showed that there were no significant differences between groups for age, body mass index, age at menarche, frequency, and duration of menstrual bleeding. The results showed that there was no significant difference between groups for the interval between the first menstruation and first dysmenorrhea (P>0.05). 

**Table 1.** Comparison of Characteristics of the Two Groups in the First Cycle ^a ^

Group	Group a (n=32) Mean ± MD	Group b (n=27) Mean ± MD	P
**Age**	21.95 ± 3.01	21.40 ± 2.45	0.631^b^
**BMI**	20.62 ± 2.60	20.20 ± 4.08	0.760^b^
**Menarche**	13.44 ± 0.98	13.04 ± 1.35	0.191^b^
**Menstrual intervalsا**	26.16 ± 4.14	26.20 ± 2.89	0.9000^b^
**Bleeding duration**	6.50 ± 1.42	6.40 ± 1.17	0.852^b^
**Family history of PD**	**No., (%)**	**No., (%)**	0.185 ^c^
Have	12 (66.7)	(45.5) 5	
Haven't	6 (33.3)	6 (54.5)	

^a^ Values are given as mean ± SD or number (percentage) unless otherwise indicated

^b^ ANOVA

^c^Pearson Chi-Square test using exact P-value

Friedman test showed that the pressure on third liver point caused pain reduction in the third cycle (after intervention) compared to the first cycle (preintervention) (Table 2). 

Table 3 shows that there was no significant difference in pain intensity between groups before and after intervention in the first and third cycles.There were no reports of adverse effects of this method of acupressure in any participant. 

**Table 2.** Comparison of Pain Median (Quartile1-Quartile3) Before and After Treatment Within Groups (Comparing Cycles 1 and 3 Within Groups)

Group	a (n = 32)				b (n = 27)			
Time From Onset of Bleeding	Cycle 1 Percentiles 50 (25-75)	Cycle 3 Percentiles 50 (25-75)	Change Percent	P^a^	Cycle 3 Percentiles 50 (25-75)	Cycle 1 Percentiles 50 (25-75)	Change Percent	P^a^
**Onset of Bleeding**	4.50 (2.00-5.00)	3.00 (1.00-6.00)	-33.33	0.257	4.00 (2.00-7.00)	4.00 (3.00 - 5.00)	0.00	1.000
**Half-Hour**	5.50 (3.00-8.00)	2.50 (1.00-6.00)	-54.54	0.028	4.00 (2.00-7.00)	6.00 (4.00 - 7.00)	-33.33	0.201
**1 Hour**	5.50 (3.00-8.00)	2.00 (1.00-6.00)	-63.63	0.008	4.00 (2.00 - 6.00)	7.00 (4.00 - 8.00)	-42.58	0.061
**2 Hour**	5.50 (3.00-8.00)	2.50 (1.00-7.00)	-54.54	0.041	4.00 (1.00 - 6.00)	7.00 (3.00 - 8.00)	-42.58	0.033
**3 Hour**	4.50 (3.00-8.00)	3.00 (1.1-6.75)	-33.33	0.001	2.00 (1.00 - 6.00)	6.00 (3.00 - 8.00)	-66.66	0.011
**4 Hour**	5.00 (2.00-8.00)	2.00 (0.00-5.00)	-60.00	0.001>	3.00 (1.00 - 6.00)	4.00 (2.00 - 6.00)	-25.00	0.513

^a^Sign Test

**Table 3.** Regression Ranks to Compare Pain Intensity Between Treatment Groups in the First and Third Cycles

Time From Onset of Bleeding	Cycles 1 Med (IQR)			Cycles 3 Med (IQR)		
	a (n = 32)	b (n = 27)	P ^a^	a (n = 32)	b (n = 27)	P ^b^
**Onset of Bleeding**	4.50 (3.00)	4.00 (2.00)	0.400	3.00 (5.00)	4.00 (5.00)	0.449
**Half-hour**	5.50 (5.00)	6.00 (3.00)	0.338	2.50 (5.00)	4.00 (5.00)	0.177
**1Hour**	5.50 (5.00)	7.00 (4.00)	0.231	2.00 (5.00)	4.00 (4.00)	0.551
**2 Hour**	7.00 (4.00)	5.50 (5.00)	0.565	2.50 (6.00)	4.00 (5.00)	0.898
**3 Hour**	4.50 (5.00)	6.00 (5.00)	0.921	3.00 (5.65)	2.00 (5.00)	0.748
**4 Hour**	5.00 (6.00)	4.00 (4.00)	0.444	2.00 (5.00)	3.00 (5.00)	0.103

^a^ Based on Mann-Whitney test

^b^ Ordinal regression analysis with adjustment for the baseline values in the first cycle

## 5. Discussion

The purpose of this study was to determine the effect of pressure on third liver point on PD. 

The age of participants in this study was similar to other studies (10, 13, 15-17).

The BMI as a confounding factor in interventional groups was not significant. Previous studies have reported conflicting results in this area. It is said that thin people have a higher risk of PD (2, 6) and it correlates with BMI (3, 8). Contradictory it was said that extra weight is an important factor for uterine cramps during the menstruation and increases the risk of prolonged pain (9). However, this variable is the same in the study groups.

In the present study, Mean ± SD age at menarche, menstrual intervals, and duration of bleeding are similar in interventional groups. Studies show that age at menarche (2, 4), menstrual intervals (5) and bleeding time (3, 5) affect PD.

In this study, family history of PD did not have a significant difference between groups (P > 0.05). This comparison was performed because other studies have shown that PD is more common in people with a family history (6, 7).

In this study there was no significant difference in pain intensity at the start of bleeding in the first and second cycles. This indicates that the two groups had similar pain before pressure. Similarly no differences were noted in the Kashefi et al. study (2010) (15).

There was significant difference in pain intensity within groups in all hours after the onset of bleeding in the third cycle compared to the first cycle in group a. Also there was a difference at 2 and 3 hours after the treatment in group b (liv3) (P < 0.05). Similarly Bazarganipour et al. (2010) showed significant difference in pain intensity with pressure on liv3 (18). Our study had different approach to pressure. In their study, pressure was performed 3-7 days prior to menstruation for 20 minutes per day, while in this study pressure was performed at the start of bleeding for less time (16 minutes). Other differences between our study and that of Bazarganipour et al. (2010) is that they applied acupressure only on the right foot for three cycles; whereas, in our study pressure was performed on both feet for two cycles. Acupressure may be more effective in the long term because in the Lin et al. study (2009) it was suggested that there is a potential to produce a long-lasting amelioration on PD (23). As can be seen, there is a decrease in pain intensity in the control group. As well some degrees of pain reduction were seen in control group in previous studies and non classical point pressing may be effective too (1, 15, 18). This effect may be due to the psychological effects. Contrary Mirbagher et al. (2010) study showed that there was no difference in pain intensity before and after intervention in control group but in their control group, placebo point received no pressure but only superficially touch (16).

This study shows that there was no significant difference in pain intensity between groups in the third cycle (P > 0.05). Probably because both groups showed pain reduction within groups, so there is no difference between them for pain relief. But in Bazarganipour et al. (2010) study differences between groups were resulted, probably because she selected people who had clinical symptoms of liver channel involvement, and pressure was applied for a longer period. Then pressure on liv3 may require a longer time to be effective. In our study, students learned the techniques to apply pressure, while in her study pressure was applied by the researcher.

Conclusions: the pressure on the third liver point applied in this investigation was effective in reducing primary dysmenorrheal pain. So using this method is recommended to reduce PD. Considering significant difference within groups, better judgment would be possible with increasing the number of participants.

Limitations: Samples of this study were students and at the time of college examination they had more tendencies to take pills for quick relief of pain. Their hard burden causes unwillingness to participate in the study and loose of samples. Also the long term of study and frequency of measurements caused loss of samples and reduction in the sample size.
